# Developing a Patient Experience Questionnaire for the Man Van Mobile Clinical Unit

**DOI:** 10.1111/hex.70443

**Published:** 2025-09-25

**Authors:** Masood Moghul, Netty Kinsella, Fionnuala Croft, Patrick Kierkegaard, Declan Cahill, Nicholas David James

**Affiliations:** ^1^ Department of Urology The Royal Marsden NHS Foundation Trust London UK; ^2^ Division of Radiotherapy and Imaging The Institute of Cancer Research London UK; ^3^ Cancer Research UK Convergence Science Centre Imperial College London and The Institute of Cancer Research London UK

## Abstract

**Introduction:**

Prostate cancer, the most common male cancer in the United Kingdom, disproportionately affects Black men and deprived communities, where early diagnosis is critical to reducing mortality. The Man Van, a mobile outreach service, targets these high‐risk groups to improve access to prostate cancer testing. The Man Van was developed to address health inequalities and other barriers to healthcare that affect prostate cancer and men's health more generally. As a novel clinical model, measuring the patients' perspectives of its quality and acceptability is relevant when evaluating its effectiveness.

**Methods:**

To facilitate evaluation of the effectiveness of the Man Van project, a novel patient questionnaire was developed using a three‐stage approach: (1) identification of domains and evaluation of existing evidence, (2) discussion groups followed by a modified virtual Delphi approach to refine themes and develop questions and (3) a real‐world evaluation to finalise the questionnaire. Virtual discussions and asynchronous feedback facilitated stakeholder input.

**Results:**

Two broad areas of acceptability and quality were identified, which were synthesised into 9 domains, linked to 15 themes to measure patients' perspectives on the Man Van. The final 17‐item questionnaire follows the patient journey from service awareness to discharge, with socio‐demographic data sourced from the Man Van database.

**Conclusions:**

This study produced the first evidence‐based questionnaire for evaluating a mobile prostate cancer outreach service, highlighting that a well‐defined service with good communication is key to establishing and maintaining quality and acceptability with patients. The tool offers a framework for evaluating and scaling similar interventions, which can be used by policymakers as a foundation for scaling up similar services.

## Patient or Public Contribution

Discussion groups that are detailed in the methods section of this paper contained patients, service users, caregivers, people with lived experience and members of the public who were key demographics for the Man Van study. These discussion groups were held in‐line with PPIE principles.

## 1 Introduction

In the United Kingdom, prostate cancer is the most common male cancer, with 50,000 cases and 15,000 deaths per year [1]. Black men are twice as likely as White men of developing the disease [2] with deprivation being a key health inequality associated with late presentation and worse outcomes [3]. Early diagnosis and treatment are key to reducing the presentation of late‐stage disease and preventing deaths. Prostate cancer cases are set to surge worldwide, and novel methods to tackle these inequalities are of increasing importance. Outreach programme initiatives to improve experience and access to healthcare to support early diagnosis [4] have been highlighted within the NHS Cancer Programme‐Faster Diagnosis Framework for exploration in relation to specific population groups with a higher risk of cancer [5].

Novel mobile outreach services, such as the Man Van developed by The Royal Marsden Hospital and Institute of Cancer Research, aim to address these disparities by delivering nurse‐led health checks, including prostate‐specific antigen (PSA) testing, to high‐risk communities (ethnic minorities and lower socio‐economic groups) [4, 6].

A pilot study demonstrated higher prostate cancer detection rates and early‐stage diagnoses compared with previous screening studies as well as higher rates of clinically significant disease, underscoring its potential [6]. When combined with the significantly higher rates of recruitment from high‐risk groups (Black men and deprived men) [6], it supports the use of the Man Van model as an effective method to level up health inequalities associated with prostate cancer. However, patient perspectives on its quality and acceptability—crucial for evaluating effectiveness and informing scale‐up—remained unexplored. We sought to address this before the Man Van being upscaled into a larger project.

Quality of care is a multidimensional concept containing both inherent characteristics of the care and outcomes [7]. The Health and Social Care Act 2012 put the patient experience as one of the three dimensions of quality, with the others being safety and clinical effectiveness [8]. Understanding patient perspectives and what they value from care quality [9] is a key element of evaluating the effectiveness of pilot projects and understanding how to improve them before wider roll‐out. Although patient perspectives are often ascertained by using satisfaction surveys [10], these can be limited in scope and detail, particularly when evaluating novel models. Satisfaction is an important, relativistic part of quality and the patient's perspective, but it is not the only part [11], and the link between them is not clear [12]. A more bespoke methodology combining qualitative and quantitative data can help to improve the depth of understanding [9] and bridge this gap.

Patient perspectives can drive outcomes important to both patients and other stakeholders [11]. Alongside quality, acceptability is another consideration which is particularly relevant to novel services and pilot projects [13].

Professional perspectives can act as a proxy for patient perspectives, but divergence between the two and the reasons behind this are important [9]. Divergence between patient perspectives versus healthcare professional perspectives can be costly to both the health system and the individual. Ensuring this divergence is limited and there are high levels of patient buy‐in with alignment of perspectives and goals is key to the success of healthcare services.

Fundamental aspects of healthcare quality were documented by the Institute of Medicine, which identified six domains: safety, effectiveness, patient‐centredness, timeliness, efficiency and equity [14]. Patients can also experience healthcare to be fragmented and lack continuity [15]. Integrated care can be used to create a sense of cohesiveness and synergy [16] and improve patient experiences. The NHS Patient Experience Framework expanded on this to define elements essential to patient experiences of NHS services: respect, co‐ordination and integration, information, physical comfort, emotional support, welcoming the involvement of family and friends, transition and continuity and access to care [17].

Patient‐reported experience measures (PREMs) are tools that can report patient satisfaction and overall experience with healthcare services [18], complementing data from clinical outcomes. Alongside PREMS, patient‐reported outcome measures (PROMS) are also used to evaluate services [9]. PREMS are focussed on patient experiences, whilst PROMs tend to measure health‐related domains [9, 18], often following administration of a drug or treatment [18]. Patient and public involvement (PPI) has also shown value in designing and delivering healthcare services and can be used in different phases of the research process [18]. Ensuring adequate understanding of patient perspectives is particularly relevant in the high‐risk groups being targeted by the Man Van: ethnic minorities and lower socio‐economic groups. These may give rise to a unique set of ‘person‐related conditions’ which can impact perceptions [19]. Factors such as medical mistrust and racism may also be relevant [11].

The Delphi approach is a commonly used method for achieving a consensus of opinion of real‐world knowledge from experts in the field. It was developed by Dalkey and Helmer at the Rand Corporation in the 1950s [20] and is aimed at problem‐solving, idea‐generation or determining priorities [21]. A modified Delphi approach can be used to develop bespoke methods of evaluating services with virtual meetings and communications speeding up the process and reducing costs [22].

We sought to develop a bespoke evaluation tool to assess the overall effectiveness (encompassing quality and acceptability) of the Man Van from patient perspectives using a modified virtual Delphi approach.

## 2 Methods

To facilitate the evaluation of the effectiveness of the Man Van project, a novel patient questionnaire was developed. Guided by a trained steering group with extensive experience of prostate cancer, consisting of a urologist (M.M.), a urology nurse consultant with expertise in qualitative research (N.K.) and a urology advanced nurse practitioner (F.C.), we conducted a three‐stage approach.

### 2.1 Stage 1: Identification of Domains and Evaluation of Existing Evidence

#### 2.1.1 Conceptual Frameworks

Domain synthesis was guided by theoretical frameworks. The Theoretical Framework of Acceptability [13] consists of seven domains that capture the psychological and practical dimensions of acceptability, key for a mobile service targeting hesitant populations and also allows for retrospective analysis. In addition to these domains, additional domains of patient expectations and patient experiences were added from the conceptual model of patients' perception of healthcare quality from Sofaer and Firminger [11]. Building on the healthcare quality framework from the Institute of Medicine [14], this model was selected for its focus on patient perceptions, distinguishing expectations (e.g., reputation of providers) and experiences, which are critical for evaluating a novel service's alignment with user needs. Its emphasis on subjective quality suited the Man Van's innovative context, where patient buy‐in is key to uptake in high‐risk groups.

#### 2.1.2 Scoping Review of Existing Evidence

A scoping review of existing literature was conducted, focussed on quality and acceptability assessment tools for clinical models, including but not limited to mobile clinics, clinics and prostate cancer. Searches of PubMed were conducted for English‐language articles, including reviews, studies and commentaries published until 1 April 2021, with no limitations on disease or patient population. Initial search terms included the following: ‘mobile clinic AND patient questionnaire’, ‘mobile clinic AND patient survey’ and ‘mobile clinic AND PREMS’. This search generated 719 articles. Further searches were conducted of the following terms: prostate cancer AND ‘patient survey’, prostate cancer and ‘patient questionnaire’. This generated 891 articles. We narrowed the list to only include articles with original research containing relevant domains with or without surveys or questionnaires relevant to understanding patient perspectives on the quality and acceptability of services/clinics in prostate cancer were selected, giving us 11 articles [23, 24, 25, 26, 27, 28, 29, 30, 31, 32, 33]. Domains and themes relevant to our model were identified from these articles.

We also reviewed pre‐existing surveys and questionnaires that were relevant to our questionnaire design. These included the following: the Consumer Assessment of Healthcare Providers and Systems Outpatient and Ambulatory Surgery Survey (OAS CAHPS) [34], the Patient Perceptions of Care Survey [35], the Massachusetts Ambulatory Care Experiences Survey [36], National Cancer Patient Experience Survey [37], Patient Satisfaction Questionnaire [38] and the quality of care from the patient's perspective questionnaire [7, 39]. We adapted domains and themes from these and developed new items to capture domains not represented in conjunction with our scoping review.

### 2.2 Stage 2: Discussion Group and Modified Virtual Delphi Approach

A virtual discussion group was led by the steering group, facilitated by one member of the steering group (N.K.) with training and prior experience in conducting discussion groups with patients. Inclusion criteria for the discussion group were patients/relatives with experience of prostate cancer or potential community stakeholders for the Man Van. Stakeholders could include but not be limited to community leaders, primary care leads and others who may be involved with the delivery of the Man Van locally. Panellists were recommended by the steering group based on and invited to participate via email along with a short video providing some background and context about the project. The video was 2 min and 42 s long and details key information about the project for stakeholders and for potential participants. It explains what tests are performed on the van as well as follow‐up procedures. The video is available at the following webpage: https://youtu.be/rWV9OYX-70Y?si=2IIx9qTOLVTZces7. Participants in the discussion group were involved based on Patient Involvement and Engagement (PPIE) principles.

During the discussion group, themes previously identified during evidence synthesis were used as discussion points, with open‐ended questions used to delve into perspectives. An aggregate analysis of open‐ended responses was performed with searching, reviewing and defining themes, with consensus of domain selection being as group solidarity or absolute alignment [40]. Question development took place following this analysis.

Following initial question formulation, a modified virtual Delphi approach [20, 22] was conducted over a 3‐week period, within the 8‐week maximum suggested timeframe [21, 41] to ensure maintenance of participant engagement. Feedback on questions was sought with an iterative consensus‐building approach using asynchronous communication via email correspondence from all participants [22].

### 2.3 Stage 3: In‐Person Real‐World Evaluation

To perform a real‐world evaluation of the finalised questionnaire, we held a further discussion group meeting with men from Man Van's target demographics: a church group within a deprived area in South London containing a predominantly Black congregation. This group reviewed all the questions for clarity, comprehensibility, understanding and use of language with the aim to finalise the structure and content of the questionnaire. Participants in this discussion group were also involved based on PPIE principles.

### 2.4 Analysis

Analysis of the results from the questionnaire will take place within a broader evaluation of the effectiveness of the Man Van. Descriptive and thematic analysis will be utilised to analyse these results within an implementation science framework.

## 3 Results

### 3.1 Evidence Synthesis

The frameworks previously mentioned identified two broad areas for our questionnaire to cover: acceptability and quality. For quality, the healthcare quality framework [11] suggests two domains: patient expectations and patient experiences of previous services. These domains infer an individual patient's criteria for quality and ultimately their perception of quality. For acceptability, the Theoretical Framework of Acceptability [13] suggests the following domains: affective attitude, burden, ethicality, intervention coherence, opportunity cost, perceived effectiveness and self‐efficacy [13]. From the literature review and pre‐existing questionnaires, we populated these domains with themes that could influence a patient's perspective. Not all domains had individual themes, and there was some overlap between these. Where themes were identified outside of the domains mentioned, additional domains were added. Nine domains were identified and linked to 15 themes as shown in Table 1.

**Table 1:** Domains, themes and aggregate analysis following the discussion group.

Domains from frameworks	Themes from evidence synthesis	Aggregate analysis following discussion group
Patient expectations [11]	Trust of organisation [25]	Importance of having trust in the team when launching a new service May be less important as NHS service and NHS branding/logos may mitigate this
	Professionalism [26] Atmosphere [29, 39] Attitude [28] and respect [36, 39]	The importance of professionalism and a light, upbeat atmosphere for promoting men's health checks in a mobile setting
	Patient demographics [27]	Not applicable as will be pre‐recorded on all patients
	Previous GP visit (possible symptoms and timing of visit) [32, 37]	Importance of finding out previous experiences of GP including whether prostate‐related symptoms were experienced, to understand pre‐existing challenges and barriers
Patient experience [11] Affective attitude [13] Intervention coherence [13] Ethicality [13] Effectiveness [13]	Global satisfaction [26, 28, 32]	Agreement on importance of having a global view of the experience
	General communication explanations [31, 34, 35, 38], results [37] Information given [28] Co‐ordination of care [33]	Importance of auditing advertisement methods, for example, word of mouth may be more relevant and difficult to measure otherwise Importance of measuring how results are delivered, for example, via telephone or face to face for bad news, with other news being communicated via letters or text messaging Importance of smooth process to communicate with GP and for onwards referrals, including timing of further cancer check appointments
	Registration process [35]	Importance of auditing registration methods Importance of checking that registration process is accessible and easy, for example, using a website for booking appointments or using a QR code may be challenging
	Time spent with patient [37, 38]	Agreed that timing should not feel rushed, optimal time
Burden [13] Opportunity cost [13] Self‐efficacy [13]	Barriers and motivations [23, 25]	Important to understand motivations for using the service
	Affordability [30]	NHS service hence free at point of use to all NHS patients
	Accessibility [30, 38] and travel distance [29]	Important to measure overall accessibility, can use comparator with GP services Van will be situated in community‐based locations, travel distance will be measured using pre‐existing demographic data, hence not needed to be measured within the questionnaire

### 3.2 Consensus Building

#### 3.2.1 Initial Discussion Group and Theme Setting

Informants during the first discussion group were four men and one woman. All participants were White, > 45 years old and users of NHS services. One man was a prostate cancer patient, and the female participant was married to a prostate cancer patient. The other three men were all stakeholders for potential Man Van collaborations as well as being potential patients. The discussion group was 100‐min long. Following the first discussion group, an aggregate analysis of the themes was performed with results shown in Table 1. A questionnaire with 14 items based on these themes and analysis was developed and is shown in Appendix 1.

#### 3.2.2 Modified Virtual Delphi Approach

The initial questionnaire was circulated to the same participants for feedback as part of the modified virtual Delphi approach. Initial discussions favoured changing questions with scoring responses from Likert scoring scales to 5‐point word scales as this may be easier to understand and give more meaningful answers. Further discussions supported including the word ‘urinary’ in Question 3 to specify the symptoms men may attend for with an example given as well. The finalised questionnaire was recirculated with unanimous agreement for the inclusion of all questions. Further detail on question development and linkage to themes is shown in Table 2.

**Table 2:** Question development and theme linkage.

Themes	Questionnaire Version 1	Review	Questionnaire Version 2
Demographics	To be included from Man Van data sets		
Previous GP experience and symptoms	Did you have any symptoms that you were worried about before coming to the Man Van?	Question accepted, but addition of word ‘urinary’ recommended to make it clearer	Did you have any problems with passing water/urination that you were worried about before coming to the Man Van? (e.g., poor flow of urine, blood in the urine or leakage)
	If you answered yes: Would you have seen your GP with any symptoms if the Man Van was not available?	Question accepted but reworded on further review	If you answered yes, did you see your GP for those symptoms?
			If you answered no, how comfortable would you be approaching your GP about them?
			How long ago did you last see your GP for reasons related to waterworks/urination?
			Was this in‐person, or via telephone or videocall?
			How long ago did you last see your GP for reasons related to waterworks/urination?
Registration	How did you book in to be seen in the Man Van?	Question accepted	How did you book in to be seen in the Man Van?
	Was it getting a Man Van appointment compared to getting a GP appointment?	Question accepted but reworded	To what degree was it easy or difficult to get a Man Van appointment compared to a GP appointment
Motivation and barriers	Not included	Domain included on further review	What motivated you to visit the Man Van?
			Would having a rectal examination in addition to a blood test for prostate cancer put you off attending a Man Van appointment?
Timing	Did you feel the length of your Man Van appointment was appropriate?	Addition of appointment length as a reminder	Did you feel the length of your Man Van appointment (15 min) was appropriate
Global satisfaction	Do you have any other comments about the Man Van?	Removed as further interviews planned	How would you rate your overall experience in the Man Van?
	Did you feel comfortable having a check‐up inside the Man Van?	Removed as encompassed by global score	
Global expectations	Did you feel the Man Van staff were professional?	Removed as encompassed by global score	Did your experience in the Man Van meet your expectations?
	Having been seen in the Man Van, would you recommend the Man Van to other men?	Question accepted but reworded to simplify	Would you recommend the Man Van to other men?
Intervention coherence and communications	How did you hear about the Man Van?	Question accepted	How did you hear about the Man Van?
	How did you receive your results following you Man Van appointment?	Question accepted, reworded	How did you receive your results following your Man Van appointment? (select all that apply)
	Were you happy at this method of receiving results?	Question accepted, reworded	Were you satisfied with quality of information given with the results?
	How else would you prefer to receive results following your appointment?	Removed as unnecessary	
Integration/co‐ordination of care	Were you referred on to any other services following your Man Van check‐up? If you answered yes: Did this referral process work well?	Split question into cancer and non‐cancer services	Were you referred to any cancer services following your Man Van appointment? If yes, did you find this to be easy and straightforward
			Were you recommended to see the GP after your Man Van appointment for any other health conditions (non‐cancer related)? If yes, did you?
Global effectiveness	Would you prefer to have access to the Man Van over the GP for a health check‐up?	Question made less antagonistic towards GPs. Addition of changes to healthcare‐seeking behaviour	If you are offered another health check for prostate cancer, are you more likely to attend the Man Van rather than your GP?
			Would you be more likely to seek healthcare advice in the future because of your experiences in the Man Van?

### 3.3 Real‐World Evaluation

The questionnaire was evaluated with five men from a predominantly Black church congregation, of which two had previously attended the Man Van. All men were of Black ethnicity and over 45 years of age from a deprived region of South London, making them a key demographic for the Man Van. All spoke fluent English. All questions were discussed and evaluated for clarity and understanding. All questions were accepted; however, the importance of ranking different answers was discussed, where more than one is given, for example, motivation factors. The finalised questionnaire had 17 items (Appendix 2), following in chronological order from the time the patient is contacted or becomes aware of the Man Van service to the point of discharge from the service.

## 4 Discussion

This study developed the first questionnaire specifically designed for a mobile prostate cancer outreach service, the Man Van, targeting high‐risk groups such as Black men and those in deprived communities. Sofaer and Firminger's model [11], which dictates patient expectations and experiences of previous services as key domains, highlights the importance of measuring the Man Van's service delivery, particularly in a novel outreach context where trust in a mobile clinic may differ from traditional settings. This was further emphasised in the discussion group feedback of trust in the NHS being a facilitator, a finding consistent with patient expectations of professionalism in established healthcare systems [42].

Domains elucidated from the Theoretical Framework of Acceptability [13], such as burden and opportunity cost, highlighted barriers relevant to high‐risk groups, such as reluctance to undergo digital rectal examinations, which emerged as a potential deterrent to attendance. This dual‐framework approach allowed us to capture both the structural aspects of quality and the psychological dimensions of acceptability, resulting in a more comprehensive evaluation than available via generic surveys. The discussion group process combined with the modified virtual Delphi approach ensured that stakeholder input moulded the questionnaire. The iterative process involved experts, patients and men from target demographics, which aligns with best practices for developing tools in health services research [43].

Our findings mirror prior research on mobile health interventions, highlighting convenience and communication as critical to patient engagement in underserved populations [44]. However, our questionnaire extends beyond existing tools by incorporating outreach‐specific domains like registration ease and motivations, which are particularly relevant for mobile services like the Man Van.

The 17‐item questionnaire, structured chronologically from service awareness to discharge, ensures a comprehensive evaluation of the Man Van's impact. Questions on registration methods and result delivery methods address practical barriers and communication preferences, which discussion groups identified as pivotal. Moreover, the inclusion of socio‐demographic data may enable future analyses of how ethnicity and deprivation influence experiences.

Our transparent study design [22] (introductory video, clear timelines and communication with participants) may contribute to high participation rates, underscoring the value of structured engagement in virtual settings. However, the initial discussion group's lack of ethnic diversity (all White participants) is a limitation, given that one of the target demographics is Black men. Although this was mitigated by the real‐world evaluation involving Black men from a deprived area, earlier input from different ethnic groups may have better captured factors such as medical mistrust, which disproportionately affects ethnic minorities [45]. Furthermore, it could have impacted the design of the questionnaire. This limitation is also insightful into the challenges of recruiting from hard‐to‐reach groups.

The small sample sizes across all stages may limit the breadth of perspectives, although smaller groups can facilitate more in‐depth discussions. The non‐anonymised nature of group meetings, while fostering collaboration, may have introduced bias, particularly among stakeholders interested in the success of the Man Van. Email feedback although not anonymous allowed for privacy in case of group bias. While the virtual modified Delphi approach may offer reduced costs and expedited consensus building, it may have excluded participants with limited digital access, relevant for deprived populations.

## 5 Conclusions

The Man Van is a novel service tackling a host of challenges in hard‐to‐reach groups of men that are high‐risk of prostate cancer. From our evaluation, it is clear that a well‐defined service with good communication is key to establishing and maintaining quality and acceptability with patients. From a rigorous three‐stage process, this questionnaire has been developed and is the first evidence‐based assessment tool for evaluating a mobile outreach service for prostate cancer. Policymakers can use findings from this questionnaire to evaluate how mobile health models can address health inequalities, particularly in hard‐to‐reach groups and provide evidence informing their scale‐up whilst also ensuring that patient perspectives remain central to their development. Given the broad nature of the domains explored in this questionnaire, the questionnaire could be adapted for other mobile units or outreach health models.

## Author Contributions

**Masood Moghul:** conceptualisation, data curation, formal analysis, investigation, resources, methodology, project administration, writing – original draft preparation, writing – review and editing. **Netty Kinsella:** data curation, formal analysis, investigation, methodology, resources project administration, supervision, writing – review and editing. **Fionnuala Croft:** data curation, investigation, resources, project administration, writing – review and editing. **Patrick Kierkegaard:** methodology, supervision, writing – review and editing. **Declan Cahill:** methodology, supervision, writing – review and editing. **Nicholas David James:** conceptualisation, methodology, project administration, supervision, writing – review and editing.

## Ethics Statement

The Man Van project has ethical approval from the Wales Research Ethics Committee (REC) 6. Study title: The Man Van Project: an evaluation of the effectiveness of a mobile outreach screening clinic model for earlier detection of prostate cancer and other male cancers. REC reference: 22/WA/0113. Protocol number: CCR5573. IRAS project ID: 304320. Discussion groups documented in this study were held in accordance with PPIE principles.

## Conflicts of Interest

The authors declare no conflicts of interest.

## Data Availability

The data that support the findings of this study are available from the corresponding author, upon reasonable request and upon consideration of any ethical/privacy concerns.
